# Analysis of Land Use/Land Cover Changes Using Remote Sensing Data and GIS at an Urban Area, Tirupati, India

**DOI:** 10.1155/2013/268623

**Published:** 2013-05-28

**Authors:** Praveen Kumar Mallupattu, Jayarama Reddy Sreenivasula Reddy

**Affiliations:** Department of Chemistry, Sri Venkateswara University, Tirupati, Andhra Pradesh 517 502, India

## Abstract

Land use/land cover (LU/LC) changes were determined in an urban area, Tirupati, from 1976 to 2003 by using Geographical Information Systems (GISs) and remote sensing technology. These studies were employed by using the Survey of India topographic map 57 O/6 and the remote sensing data of LISS III and PAN of IRS ID of 2003. The study area was classified into eight categories on the basis of field study, geographical conditions, and remote sensing data. The comparison of LU/LC in 1976 and 2003 derived from toposheet and satellite imagery interpretation indicates that there is a significant increase in built-up area, open forest, plantation, and other lands. It is also noted that substantial amount of agriculture land, water spread area, and dense forest area vanished during the period of study which may be due to rapid urbanization of the study area. No mining activities were found in the study area in 1976, but a small addition of mining land was found in 2003.

## 1. Introduction

In an urban environment natural and human-induced environmental changes are of concern today because of deterioration of environment and human health [1]. The study of land use/land cover (LU/LC) changes is very important to have proper planning and utilization of natural resources and their management [2]. Traditional methods for gathering demographic data, censuses, and analysis of environmental samples are not adequate for multicomplex environmental studies [3], since many problems often presented in environmental issues and great complexity of handling the multidisciplinary data set; we require new technologies like satellite remote sensing and Geographical Information Systems (GISs). These technologies provide data to study and monitor the dynamics of natural resources for environmental management [4]. 

Remote sensing has become an important tool applicable to developing and understanding the global, physical processes affecting the earth [5]. Recent development in the use of satellite data is to take advantage of increasing amounts of geographical data available in conjunction with GIS to assist in interpretation [6]. GIS is an integrated system of computer hardware and software capable of capturing, storing, retrieving, manipulating, analyzing, and displaying geographically referenced (spatial) information for the purpose of aiding development-oriented management and decision-making processes [7]. Remote sensing and GIS have covered wide range of applications in the fields of agriculture [8], environments [9], and integrated eco-environment assessment [10]. Several researchers have focused on LU/LC studies because of their adverse effects on ecology of the area and vegetation [11–14].

**Figure 1:**
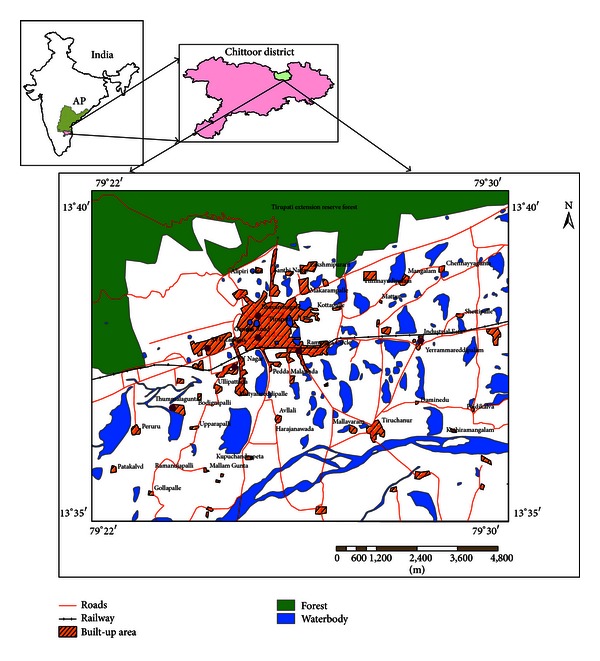
Location of the study area, Tirupati.

Present study area witnessed rapid development during past decades in terms of urbanization, industrialization, and also population increase substantially. The main objective of this paper is to detect and quantify the LU/LC in an urban area, Tirupati (Figure 1), from 1976 to 2003 using satellite imagery and topographic map. 

## 2. Study Area Description 

The study area, Tirupati region (Figure 1), is located nearby the metropolitan city, Chennai, at a distance of about 145 km in southern peninsular India. Tirupati is a world famous holy pilgrim place for devotees of Lord Sri Venkateswara is situated in Chittoor district of Andhra Pradesh (AP) state at an altitude of 182.9 m (13.05°N latitude and 79.05°E longitude) which represents an urban area surrounded by major industrial and agricultural activities along with dense forest. The town area owes its existence to the sacred world famous temple of Lord Sri Venkateswara situated on the seven hills (Tirumala) adjoining it. The total population of Tirupati region is about 3, 09,000 according to 2001 census of India. Industrial activities have also impact on the overall pollution levels. The major industries are located heavily at Tirupati industrial area situated at the east nearby Renigunta.

The study area covers many water streams, majorly the Swarnamukhi River basin. All the streams including the Swarnamukhi River are ephemeral and rise from the Tirupati hill ranges. The annual rainfall during the study period is 899.8 mm with total number of 43 events, in which the highest rainfall in July (340.6 mm) and the lowest in April (5.6 mm). The streams, while flowing from the upland to lowlands, form steeply dissected valleys often covered with boulders, showing striations. The surface runoff in most of the streams is restricted to a few hours after the rain, while in the Swarnamukhi and Rallakalva Rivers, the flows last for a few days to a few weeks after the rain. Most of the year, they are dry.

## 3. Data and Methodology

In the present study we have used mainly two types of data. These are topographic map and remote sensing data. The remote sensing data of georeferenced and merged data of LISS III and PAN of IRS ID of 2003 in the digital mode are obtained from the National Remote Sensing Agency (NRSA), Government of India, Hyderabad, and used. The spatial resolutions of LISS III and PAN are 23.5 and 5.8 meters, and spectral resolutions are 4 and 1 meters, respectively. 

The topographic map 57 O/6 (1:50,000 scale) is obtained from the Survey of India, Hyderabad, which was surveyed and prepared in 1976; it is converted to digital mode using scanning. The topographic map is georeferenced with longitude and latitudes using the ArcGIS software and spatial analyst tools and demarcated the boundary of study area.

A supervised signature extraction with the maximum likelihood algorithm was employed to classify the digital data of IRS 1D georeferenced and merged LISS III and PAN for land use/land cover mapping for the year 2003. Before the preprocessing and classification of satellite imagery began, an extensive field survey was performed throughout the study area using Global Positioning System (GPS) equipment. This survey was performed in order to obtain accurate locational point data for each land use and land cover class included in the classification scheme as well as for the creation of training sites and for signature generation. 

The satellite data was enhanced before classification using histogram equalization in ERDAS Imagine 8.7 to improve the image quality and to achieve better classification accuracy. In supervised classification, spectral signatures are developed from specified locations in the image. These specified locations are given the generic name “training sites” and are defined by the user. Generally a vector layer is digitized over the raster scene. The vector layer consists of various polygons overlaying different land use types. The training sites will help to develop spectral signatures for the outlined areas.

**Figure 2:**
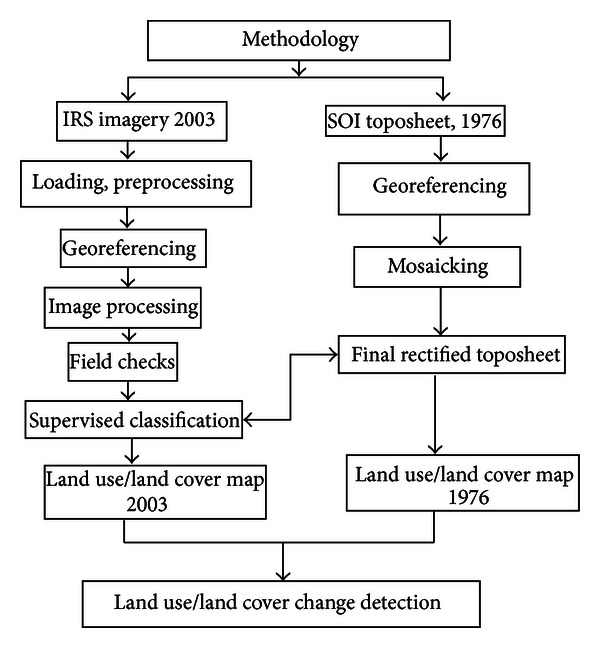
Flow chart of methodology for land use/land cover and change detection.

The land use maps pertaining of two different periods were used for postclassification comparison, which facilitated the estimation of changes in the land use category and dynamism with the changes. Postclassification comparison is the most commonly used quantitative method of change detection [15–17] with fairly good results. Postclassification comparison is sometimes referred to as “delta classification” [18]. It involves independently produced spectral classification results from different data sets, followed by a pixel-by-pixel or segment-by-segment comparison to detect changes in the classes. The detailed methodology adopted was given in Figure 2.

## 4. Results and Discussion

Knowledge about land use/land cover has become important to overcome the problem of biogeochemical cycles, loss of productive ecosystems, biodiversity, deterioration of environmental quality, loss of agricultural lands, destruction of wetlands, and loss of fish and wildlife habitat. The main reason behind the LU/LC changes includes rapid population growth, rural-to-urban migration, reclassification of rural areas as urban areas, lack of valuation of ecological services, poverty, ignorance of biophysical limitations, and use of ecologically incompatible technologies.

Present study area Tirupati is a rapid developing town and is a world famous pilgrim centre for the devotees of Lord Sri Venkateswara. During the past few decades, the study area has witnessed substantial increase in population (Table 1), economic growth, and industrialization, and transportation activities (Table 1) have negative impact on the environmental health of the region. 

**Figure 3:**
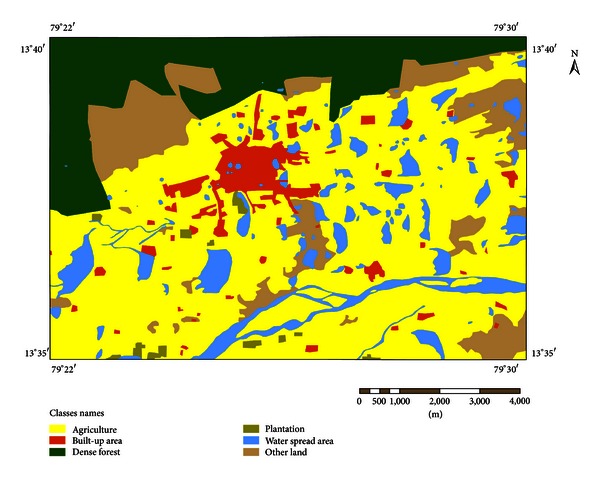
Land use/land cover in 1976.

**Figure 4:**
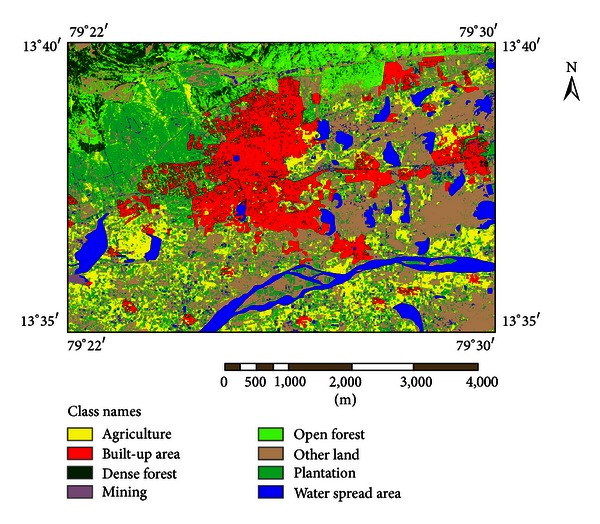
Land use/land cover in 2003.

Due to involvement of multiple data sets, we used latest technologies like remote sensing and GIS to quantify LU/LC. On the basis of interpretation of remote sensing imagery, field surveys, and existing study area conditions, we have classified the study area into eight categories, that is, agriculture, built-up area, dense forest, mining, open forest, other land, plantation, and water spread area (Figures 3 and 4). The study area covers 125 km^2^ and LU/LC changes were estimated from 1976 to 2003.

Table 2 gives the statistical results of LU/LC changes. It is evident from Table 2 that the LU/LC changes were of highest amount in agriculture built-up area, plantation, other land, and dense forest from 1976 to 2003. Comparison of LU/LC in 1976 and 2003 derived from toposheet and satellite imagery interpretation indicates that the built-up area, comprising human habitation developed for nonagricultural uses like building, transport, and communications is largely broadened from 5.91 km^2^ (1976) to 18.34 km^2^ (2003), with a net addition of 12.44 km^2^. This is due to urban expansion and population increase in this study area during the study period. 

The agricultural lands which are used for paddy and production of food, vegetables, and other mixed varieties like mango, coconuts, and other homestead trees are largely decreased from 68.23 km^2^ (1976) to 21.45 km^2^ (2003), with net decrease of 46.78 km^2^.

The study area witnessed large amount of agriculture land converted into settlements and other urban development activities. Water spread area, both man-made and natural water features such as rivers/streams, tanks, and reservoirs, also decreased from 12.09 km^2^ in 1976 to 9.91 km^2^ in 2003, with net decline of 2.18 km^2^. Water spread area decrease is occurred due to the gradual conversion of water spread area into built-up area or human developmental area as the population increased significantly during the past decades. Dense forest comprising all land with tree cover of canopy density of 70% and above is significantly declined from 1976 (22.35 km^2^) to 2003 (4.25 km^2^) with a net decrease of 18.10 km^2^. This is attributed to conversion of forest lands into urban areas and other development activities. 

Open forest land comprising all lands with tree cover of canopy density between 10% and 40% is not found in 1976, whereas there is a significant addition of 10.90 km^2^ of land in 2003 which is due to implementation of afforestation works by Tirupati municipality during the period of 2001–2003 under Haritha project (http://www.tirumala.org/activities_social_haritha.htm). The plantation land which includes agricultural tree crops and other horticulture nurseries also increased from 0.79 km^2^ (1976) to 21.80 km^2^ (2003), with a net increase of 21.01 km^2^. The other land consisting of roads, mostly link roads, joining the village settlement and barren land with or without scrub and sandy area is largely broadened from 15.64 km^2^ (1976) to 38.22 km^2^ (2003) with a net increase of 22.54 km^2^. In 1976 no mining activities were found in the study area, but a small addition of 0.13 km^2^ mining land was found in 2003.

## 5. Conclusions

This paper focuses on LU/LC changes in an urban area, Tirupati, India, using remote sensing data and GIS technology. Our results clearly show that LU/LC changes were significant during the period from 1976 to 2003. There is significant expansion of built-up area noticed. On the other hand there is decrease in agricultural area, water spread area, and forest areas. This study clearly indicates the significant impact of population and its development activities on LU/LC change. This study proves that integration of GIS and remote sensing technologies is effective tool for urban planning and management. The quantification of LU/LC changes of Tirupati area is very useful for environmental management groups, policy makers and for public to better understand the surrounding.

## Figures and Tables

**Table 1:** Study area population and vehicle fleet*.

Year	Population	Year	Vehicles
1981	273540	1990	16270
1991	372045	2000	55503
2001	309000	2003	80024

*Source: Tirupati Urban Development Authority, Tirupati.

**Table 2:** Land use/land cover changes from 1976 to 2003.

Class name	Area, km^2^		Change km^2^
	1976	2003	
Built-up area	5.91	18.34	12.43
Agriculture	68.23	21.45	−46.78
Water spread area	12.09	9.91	−2.18
Dense forest	22.35	4.25	−18.10
Open forest	0.00	10.90	10.90
Plantation	0.79	21.80	21.01
Other land	15.64	38.22	22.57
Mining	0.00	0.13	0.13

Total	125.00	125.00	0.00

(−) indicates decrease.
